# Improving management of patients with life-threatening emergency conditions in the emergency department of Sant’Andrea Hospital Rome, Italy

**DOI:** 10.1186/1472-6963-14-S2-P28

**Published:** 2014-07-07

**Authors:** Assunta De Luca, Gianluca D’Agostino, Cristiana Luciani, Domenico Antonio Lentile, Francesco Stella

**Affiliations:** 1 Health Direction- Hospital Sant’Andrea II “Sapienza” University of Medicine and Psychology, Rome, Italy; 2 Health Care Quality Unit- Hospital Sant’Andrea II “Sapienza” University of Medicine and Psychology, Rome, Italy

## Background

The Hospital practices the Clinical Governance approach to ensure high standards of care and to continuously improve the quality of services. One purpose is to produce and to apply Organizational and Clinical Procedures (OCP), based on the best available evidence and the local contest and organization, to manage critical patients in the Emergency Department (ED). Annually, about 50.000 patients arrived to the ED, where 18% have urgent and 2% life-threatening conditions. These last cases receive a multidisciplinary treatment (emergency physicians, anaesthesiologists) and after stabilization of main symptoms are delivered to the Intensive Care Unit (ICU). The emergency physician is in the permanent staff of the ED while the anaesthesiologist of the ICU is on call.

Until 2011, the anaesthesiologist was called by the emergency physician after his first intervention on the critical patient’s ED needed resuscitation treatment with consequent delay of patient’s delivery to the ICU.

In 2012, to reduce the time of intervention of the anaesthesiologist in the ED, a group was constituted by health personnel of the ED and ICU, health direction, Quality Unit. The group detected the causes of the delay through the study of available data extracted by the ED electronic data sheet and of the organization of the ED. The group decided to provide an OCP to manage patients with life-threatening emergency conditions in the ED (OCP-ED).

## Materials and methods

To reduce obstacles to the implementation and acceptance of organizational change, the emergency physicians and anaesthesiologists were educated to use OCP-ED applying the concepts and tools of experiential learning. Periodically, health direction organized meetings with personnel to disseminate the results of OCP-ED’s implementation and to discuss the possible problems and to find the solutions to overcome them. The effects of OCP-ED are measured through the indicators and the periodical audit on cases. The process indicators are calculated on data extracted by health database of the ED.

## Results

Participants positively evaluated both the educational programme and the organizational and clinical indications of the OCP-ED. It has facilitated the communication between the emergency physician and the anaesthesiologist and reduced the delay to ICU delivery.

## Conclusion

The application of the OCP-ED helped standardize behaviours in an environment characterized by great professional heterogeneity. All health personnel are learning to use the clinical and non-clinical data, periodically reviewing the cases to monitor their activities and then to improve the quality of care both in terms of speed response and appropriate therapy uses.

